# Disease burden among migrants in Morocco in 2021: A cross‑sectional study

**DOI:** 10.1371/journal.pone.0281129

**Published:** 2023-01-27

**Authors:** Firdaous Essayagh, Touria Essayagh, Meriem Essayagh, Mohammed Khouchoua, Hajar Lemriss, Mourad Rattal, Germain Bukassa, Sanah Essayagh

**Affiliations:** 1 Faculté des Sciences juridiques, économiques et sociales, Laboratoire Droit privé et enjeux de développement, Université Sidi Mohamed Ben Abdellah, Fès, Morocco; 2 Laboratoire Sciences et Technologies de la Santé, Hassan First University of Settat, Institut Supérieur des Sciences de la Santé, Settat, Morocco; 3 Office Nationale de Sécurité Sanitaire des produits Alimentaires, Oriental, Morocco; 4 Délégation de la Santé, Meknès, Morocco; 5 Department of Indigenous Services Canada/Government of Canada, Health Surveillance and Assessment Unit, First Nations and Inuit Health, Regina, SK, Canada; 6 Faculté des Sciences et Techniques, Laboratoire Agroalimentaire et Santé, Hassan First University of Settat, Settat, Morocco; UNITED STATES

## Abstract

**Background:**

Morocco, traditionally an emigration country, has evolved into not only a transit country to Europe but also a country of residence for an increasing number of migrants, with 102,400 migrants in 2019. This is due to its geographic location, the induced effects of its "African policy," and the various laws adopted by Moroccan legislators in recent years. The purpose of this study is to determine the prevalence of communicable and noncommunicable diseases among migrants such as Hepatitis C virus (HCV), human immunodeficiency virus (HIV), diabetes, and hypertension.

**Methods:**

We conducted a cross-sectional study in Oujda, Morocco, between November and December 2021. Face-to-face interviews with enrolled migrants aged 18 years and over, present in Oujda and attending an association, were carried out to collect socio-demographic data, lifestyle behaviors, and clinical parameters. Diabetes and hypertension were the primary outcomes. The Pearson’s chi-squared test and the student’s t-test were used to assess the bivariate associations between primary outcomes and categorical and continuous variables. In a multivariate model, we adjusted for predictors that were significant (p-value ≤0.05) in bivariate analysis to estimate Adjusted Odd Ratios (AOR) and 95% confidence intervals (CI).

**Results:**

There were 495 migrants enrolled, with a male/female ratio of two and an average age of 27.3±11.5 years (mean±standard deviation), ranging from 18 to 76 years. Hepatitis C virus, human immunodeficiency virus, diabetes, and hypertension were found in 1%, 0.2%, 3.8%, and 27.7% of the population, respectively. Family history of diabetes was a risk factor for diabetes in the Oujda migrant population, with an Adjusted Odds Ratio (AOR) of 5.36; CI% [1.23–23.28]. Age (AOR of 1.1; CI% [1.06–1.13]) and African origin (AOR of 3.07; CI% [1.06–8.92]) were identified as risk factors for hypertension.

**Conclusion:**

Migrants in Oujda are healthy. The high prevalence of hypertension, as well as the presence of HCV and HIV positive cases, emphasizes the importance of routine screening for hypertension, HCV, and HIV in order to detect and treat these diseases as early as possible.

## Introduction

Economic needs, illegal migration networks, climate change and natural disasters, and human conflicts all drive global migration. Global estimates place the number of international migrants in 2020 at 281 million [1]. Morocco, traditionally an emigration country, has evolved into not only a transit country to Europe but also a destination for an increasing number of immigrants, refugees, and asylum seekers. Morocco recorded 102,400 migrants in 2019, with the vast majority coming from Sub-Saharan Africa, Syria, and Yemen [1]. Morocco has twelve administrative regions, three of which are mainly concerned with the reception of a large number of migrants, namely the region of Rabat-Salé-Kénitra, the region of Tangier-Tétouan-Al Hoceima, and the region of Oriental. The strategic geographical position of the eastern region, which borders Europe to the north and the Algerian border to the east, serves as a crossroads for communication and Maghreb and European exchanges and as a link between Africa and Europe [2]. The Oujda prefecture is one of the seven provinces that make up the Oriental region. It is marked by the presence of associations very popular with migrants, refugees, and asylum seekers, including those who have recently arrived [3].

Migrants present a heterogeneous group with health needs different from those of the populations of the host countries. The pre-migration characteristics of their countries of origin, where certain transmissible diseases are present in an endemic situation such as Hepatitis C virus (HCV) and human immunodeficiency virus (HIV) and the post-migration characteristics marked by a difficult psychosocial environment, a limitation in access to preventive and curative medicine, overcrowding, unsanitary housing, and limited access to healthy food all increase their risk of contracting diseases [4].

Data from an epidemiological survey carried out in 2018 in the Rabat-Salé-Kénitra region among 25 associations working for the health of migrants and people in vulnerable situations showed that 50.0% of their health interventions with the migrant population are oriented towards sexually transmitted diseases, 8% towards cardiovascular diseases including high blood pressure, and 6.0% towards diabetes [5].

The 2013 survey in Morocco in Rabat, among 687 sub-Saharan migrants in an irregular administrative situation, showed a prevalence of sexually transmitted diseases of 7.1% [6].

Late diagnosis of these diseases among migrants can result in a significant economic burden for individuals and the public health system due to the complications associated with these diseases and the expensive drugs used to treat them.

In Morocco, a lack of data on migrants’ health, insufficient or incomplete data reported from epidemiological surveillance systems, and a scarcity of epidemiological surveys may jeopardize the migrant population’s health and quality of life. The purpose of this research was to describe the epidemiology of these diseases among Morocco’s migrant population in order to address the situation and prevent the spread of HCV, HIV, diabetes, and hypertension. An improved understanding of the prevalence of these diseases will aid in the implementation of public health policies aimed at improving migrants’ health and easing their integration into Moroccan society.

## Methods

### Study design and population

We conducted a cross-sectional study from November to December of 2021 in Oujda, Morocco among migrant population frequenting associations. We used a two-stage sampling method. The primary unit was made up of associations that help people in vulnerable situations. The secondary unit consisted of (1) migrants aged 18 and up, (2) present in Oujda, and (3) attending an association where the survey is held who agreeing to participate in the study. A simple drawing was made to select seventeen primary units from the 30 associations in Oujda. A random draw of the order numbers of the migrants was conducted in each chosen primary unit to determine the 25 participants to be included. Pregnant women were excluded from the study.

### Sample size determination

We determined the minimum sample size for HCV and HIV to be 96 migrants based on a 95% confidence interval, a 6% HCV and HIV prevalence estimate, a 5% margin of error, and 10% inflation.

We determined the minimum sample size for diabetes and hypertension to be 423 migrants based on a 95% confidence interval, a 50% prevalence of diabetes and hypertension, a 5% margin of error, and 10% inflation.

### Data collection

Staff trained in the study approached potential participants on the premises of the selected associations, briefly explaining the study and inviting them to participate. After a brief discussion with potential participants, people who met the inclusion criteria and provided consent were invited to a private room for a face-to-face interview and clinical and paraclinical data collection. During the interview with the participant, we used a standardized questionnaire to collect the following data: socio-demographic background, lifestyle and behavioral habits, and clinical parameters. Screening for the various diseases studied was carried out at the end of the interview. The same questionnaire and measuring devices (blood pressure monitors, personal weight scales, height rods, measuring tape, glucometers, screening kits, and confirmation tests) were used to limit measurement bias, and the same trained investigators nurses were involved. Participants were not compensated in order to reduce selection bias. To ensure confidentiality and anonymity, the data was collected in a private room. The survey was conducted throughout the week, including Saturdays and Sundays, to limit healthy worker bias. It was made clear to participants that if they were diagnosed with or suspected of having any medical condition, this information would be kept private. As a result, their legal status or job eligibility in Morocco would be unaffected.

Rapid screening tests allowed for on-site communication of results. Participants who were diabetic or hypertensive were referred to primary health care facilities for free management. Participants who tested positive for HIV and/or HCV were referred to Oujda’s infectious disease referral center for confirmation and management. The referral center for infectious diseases in Oujda treated confirmed HIV cases for free.

For HCV, the reference center for infectious diseases provided assistance to those who benefit from the Moroccan Ministry of Health’s medical assistance regime. The research team provided HCV management to those without medical coverage. All treatments are provided at no cost to participants.

### Definition of variables

#### Diseases screening

**Hepatitis C virus (HCV) infection** was screened by an immunochromatographic rapid test (SD Bioline HCV Test) and/or self-reported HCV. We confirmed positive results using a plasma RNA assay.

**HIV infection** was screened for by the HIV 1/2/O Tri-line Human Immunodeficiency Virus Rapid Test Device (Abon Biopharm) and/or self-reported HIV. ELISA tests confirmed the positive results.

**Diabetes mellitus** was defined as fasting plasma glucose levels greater than 126 mg/dL, blood glucose levels greater than 200 mg/dL at any time of day, or self-reporting diabetes [7].

Participants who stated they were diabetic were asked whether they were receiving pharmacological or non-pharmacological treatment.

**Blood pressure** was monitored using an electronic sphygmomanometer (Omron M2 with an accuracy of±3 mmHg). Investigators used a cuff adjusted to the participant’s arm. At the end of the interview, we measured blood pressure after about 25 minutes of rest while a chair in the sitting position supported the participant. The participant’s arm was supported at the height of the heart, feet on the ground, and his or her legs were not crossed; the participant was not allowed to talk during the measurement. To determine which arm had the highest blood pressure, a first blood pressure reading was taken on each arm. Two additional measurements were taken on the high blood pressure arm, with a one-minute interval between them. To eliminate the white coat effect, the investigator left the participant alone in the room at the end of the first measurement. The participant’s blood pressure was calculated by taking the average of the two last measurements.

The European Society of Hypertension and the European Society of Cardiology (ESH/ESC) guidelines were used to classify hypertension. This guideline defines hypertension as systolic blood pressure of 140 mmHg or higher and/or diastolic blood pressure of 90 mmHg or higher, as well as self-reported hypertension [8–10]. Participants who stated they were hypertensive were asked if they were on pharmacological or non-pharmacological treatment and were managed accordingly.

Diabetes and hypertension were the primary outcome in this study. Other’s variables were included like of socio-demographic data, lifestyle behaviors, and clinical parameters.

Marital status was classified into two categories: (1) partnered if the person is married or in a cohabiting relationship, and (2) single if the person is single, divorced, or widowed.

Migrants were defined as any foreign-born individual, with or without legal status in Morocco, regardless of their date of entry and the length of their stay, or even settlement [6]. Migrants’ legal status was divided into four categories: (i) regular legal status with legal status in the host country; (ii) irregular legal status without legal status in the host country; (iii) asylum seekers seeking safety from persecution or harm in a country other than their own and awaiting a response to their refugee claim; and (iv) refugees unable or unwilling to return to their country of origin due to a well-founded fear of persecution [11].

The country of origin of migrants has been divided into two: those from Sub-Saharan African countries and those from the Eastern Mediterranean Region. Sub-Saharan Africa included: Burkina Faso, Burundi, Cameroon, Comoros, Congo, Ivory Coast, Eritrea, Gabon, Gambia, Guinea, Guinea-Bissau, Mali, Nigeria, Central African Republic, Senegal, Serra-Lion, Sudan, Chad, and Togo, while the Eastern Mediterranean Region included only Syria.

Housing type was classified into two categories: (1) house, referring to living in a house, apartment, or reception center, and (2) homeless, referring to living in the street.

Comorbidity defined as every participant who present heart disease, dyslipidemia, or chronic renal failure. The comorbidity was classified into two categories: (1) yes or (2) no.

Overweight and obesity were defined according to the WHO classification of the body mass index. It was classified into two categories: (1) yes or (2) no [9, 12].

### Statistical analysis

All precautions were taken to ensure the anonymity of the participants and the confidentiality of the information gathered. Epi Info version 7.2.0.1 was used for data analysis. The significance level was set at 0.05, and all tests were bilateral. For continuous variables, we used mean±standard deviation, and for categorical variables, we used percentages.

We performed a simple descriptive analysis on the entire study population, followed by a subgroup analytical analysis. The Pearson’s chi-squared test was used to assess the relevance of categorical variables, while the student’s t-test was used to assess the relevance of continuous variables. A multivariate logistic regression analysis identified risk factors for diabetes and hypertension. The association between each assumed risk factor and each disease was estimated using the odds ratio and its 95% confidence interval.

### Ethical considerations

Following information and an explanation of the study’s objectives, all participants in the study were asked to sign a statement of consent in accordance with the Helsinki Declaration. The collected information’s contents and processing were handled anonymously. The study protocol #03/21 was reviewed and approved by the ethical review board of the University of Moulay Ismail of Meknes in Morocco.

## Results

### Socio-economic and demographic characteristics

We screened 495 migrants for diabetes, hypertension, and HIV; 19 (3.8%) were diabetics, 137 (27.7%) were hypertensive, and one (0.2%) was HIV positive. For HCV, 495 people were asked about their serological status and 100 were tested. HCV was detected in one case (1%). The male/female sex ratio was 2.2, and the average age ranged from 18 to 76 years. Single migrants made up 347 (70.1%), those of Sub-Saharan origin made up 389 (78.6%), those in an irregular legal status made up 228 (46.1%), and those staying in Morocco for less than five years made up 382 (77.2%) “Table 1”.

**Table 1:** The socio-economic and demographic characteristics of the migrant population in Oujda, Morocco, in 2021.

		Diabetes	*p-value*	Hypertension	*p-value*
Participants’ total no. (%)	495	19 (03.8)		137 (27.7)	
Age in years±sd	28.0±10.9	43.3±20.3	0.0036	33.7±13.9	<0.001
**Variables no. (%)**					
Age group in years (n = 495)			<0.001		<0.001
≥40	65 (13.1)	11 (16.9)		35 (53.8)	
[29–39]	93 (18.8)	01 (01.1)		39 (41.9)	
[18–28]	337 (68.1)	07 (02.1)		63 (18.7)	
Sex (n = 495)					
Male	343 (69.3)	10 (02.9)	0.10	95 (27.7)	0.98
Female	152 (30.7)	9 (05.9)		42 (27.6)	
Marital status (n = 495)			0.08		<0.001
Partnered^†^	148 (29.9)	9 (06.1)		62 (41.9)	
Single^‡^	347 (70.1)	10 (02.9)		75 (21.6)	
Education (n = 495)			0.23		0.12
Illiterate	113 (22.8)	8 (07.1)		29 (25.6)	
Elementary	146 (29.5)	6 (04.1)		52 (35.6)	
Middle school	59 (11.9)	2 (03.4)		14 (23.7)	
High school	97 (19.6)	2 (02.1)		21 (21.6)	
College	80 (16.2)	1 (01.2)		21 (26.2)	
Native country (n = 495)			<0.001		<0.001
Sub-Saharan Africa	389 (78.6)	7 (01.8)		91 (23.4)	
Eastern Mediterranean Region	106 (21.4)	12 (11.3)		46 (43.4)	
Duration of stay in Morocco (in years) (n = 495)			0.13		0.001
≥5	113 (22.8)	7 (06.2)		45 (39.8)	
<5	382 (77.2)	12 (03.1)		92 (24.1)	
Legal status (n = 495)			0.0005		0.0006
Irregular	228 (46.1)	10 (04.4)		83 (36.4)	
Asylum seeker	177 (35.8)	2 (01.1)		32 (18.1)	
Refugee	40 (08.1)	6 (15.0)		9 (22.5)	
Regular	50 (10.1)	1 (02.0)		13 (26.0)	
Number of countries crossed (n = 459)			0.002		<0.001
≥3	144 (31.4)	11 (07.6)		91 (63.2)	
<3	315 (68.6)	6 (01.9)		46 (14.6)	
Housing type (n = 495)			0.30		0.08
House*	386 (78.0)	13 (03.4)		114 (29.5)	
Homeless	109 (22.0)	06 (05.5)		23 (21.1)	
Number of persons per house (n = 386)			0.57		0.12
≥10	42 (10.9)	2 (04.8)		7 (16.7)	
[5–9]	203 (52.6)	8 (03.9)		66 (32.5)	
≤4	141 (36.5)	3 (02.1)		42 (29.8)	
Occupation (n = 495)			0.93		0.76
No	471 (95.1)	18 (3.8)		131 (27.8)	
Yes	24 (04.9)	1 (04.2)		6 (25.0)	
Monthly income ($) (n = 495)			0.18		0.14
≤150	315 (63.6)	15 (04.8)		81 (25.7)	
[150–200]	115 (23.3)	4 (03.5)		40 (34.8)	
>200	65 (13.1)	0 (00.0)		16 (24.6)	
Health insurance (n = 495)			0.72		0.28
No	492 (99.4)	19 (03.9)		137 (27.8)	
Yes	3 (00.6)	0 (00.0)		0 (00.0)	

sd stands for standard deviation.

*: House refers to living in a house, apartment, or reception center.

^†^: Partnered refers to being married or in a concubine relationship.

^‡^: Single means single, divorced, or widowed.

When the conditions were valid, the Pearson chi-2 test estimated the association between the dependent variable and the independent variables. We used a comparison test of two means for the quantitative variables; a p-value less than 0.05 was considered significant.

### Behavioral and clinical characteristics

According to behavioral data analysis, 102 (20.6%) of participants used tobacco, 83 (16.8%) drank alcohol, and 194 (39.2%) were overweight or obese. The presence of a family history of diabetes and hypertension was found in 12.9% and 20.0% of the population, respectively “Table 2”.

**Table 2:** Behavioral and clinical characteristics of the migrant population, Oujda, Morocco, 2021.

	n = 495	Diabetes	*p-value*	Hypertension	*p-value*
	no. (%)	(n = 19)		(n = 137)	
Tobacco consumption			0.07		0.05
Yes	102 (20.6)	7 (06.9)		20 (19.6)	
No	393 (79.4)	12 (03.1)		117 (29.8)	
Alcohol consumption			0.17		0.04
Yes	83 (16.8)	1 (01.2)		15 (18.1)	
No	412 (83.2)	18 (04.4)		122 (29.6)	
Physical activity			0.08		0.66
Unsatisfactory	48 (09.7)	4 (08.3)		12 (25.0)	
Satisfactory	447 (90.3)	15 (03.3)		125 (28.0)	
Stress			0.32		0.07
Yes	361 (72.9)	12 (03.3)		92 (25.5)	
No	134 (27.1)	7 (05.2)		45 (33.6)	
Salty diet			0.08		0.07
Salty	277 (56.0)	7 (02.5)		68 (24.5)	
Without	218 (44.0)	12 (05.5)		69 (31.6)	
Family history of diabetes			<0.001		0.83
Yes	64 (12.9)	10 (15.6)		17 (26.5)	
No	431 (87.1)	9 (02.1)		120 (27.8)	
Family history of hypertension			0.002		0.004
Yes	99 (20.0)	9 (09.1)		16 (16.1)	
No	396 (80.0)	10 (02.5)		121 (30.6)	
Comorbidity			<0.001		0.47
Yes	17 (03.4)	4 (23.5)		6 (35.3)	
No	478 (96.6)	15 (03.1)		131 (27.4)	
Overweight/obesity			0.01		0.004
Yes	194 (39.2)	13 (06.7)		69 (35.6)	
No	301 (60.8)	6 (02.0)		68 (22.6)	
HIV			0.84		NS
Yes	1 (00.2)	0 (00.0)		0 (00.0)	
No	494 (99.8)	19 (03.8)		137 (27.7)	
HCV (n = 100)			NS		NS
Yes	1 (01.0)	0 (00.0)		0 (00.0)	
No	99 (99.0)	3 (03.0)		25 (25.3)	

Comorbidity stands for heart disease, dyslipidemia or chronic renal failure

HCV stands for Hepatitis C Virus.

HIV stands for human immunodeficiency virus.

NS stands for non-significate.

When the conditions were valid, the Pearson Chi-2 test estimated the association between the dependent variable and the independent variables; a p-value less than 0.05 was considered significant.

### Hepatitis C infection

One of the 100 who were randomly tested for HCV was positive. He was a 55-year-old widowed asylum seeker, originating from Ivory Coast; he had been living in Morocco for the last four years, earning between $300 and $499 per month. The participant stated that he had never used injection drugs or shared sharp objects with others. In addition, the participant had viral hepatitis B and cardiovascular disease.

### HIV infection

One of the 495 participants tested positive for HIV. She was a 24-year-old illegal migrant from Ivory Coast living in a concubinage after crossing Mali and Algeria to arrive in Morocco a fortnight ago. The participant is illiterate. She had her first sexual encounter at the age of 17, and she has had over four sexual partners since then. She admitted to using condoms during sexual encounters with other partners on occasion. She declared that she practiced transactional sex with her last partner. She denied injecting drugs or sharing sharp objects with others. She admitted that she had gotten a tattoo in the previous six months.

### Diabetes

Diabetes was diagnosed in 19 of the 495 participants, representing a 3.8% prevalence. The average age of diabetics in the migrant population was 43.3±20.3 years with a male/female ratio of 1.1. Among the diabetic migrants, 12 (63.1%) were from the Eastern Mediterranean Region and 10 (52.6%) were in irregular legal status “Table 1”. The behavioral characteristics revealed that 7 (36.8%) used tobacco, 13 (68.4%) were overweight or obese, and 10 (52.6%) had a family history of diabetes “Table 2”.

### Hypertension

**Fig 1:**
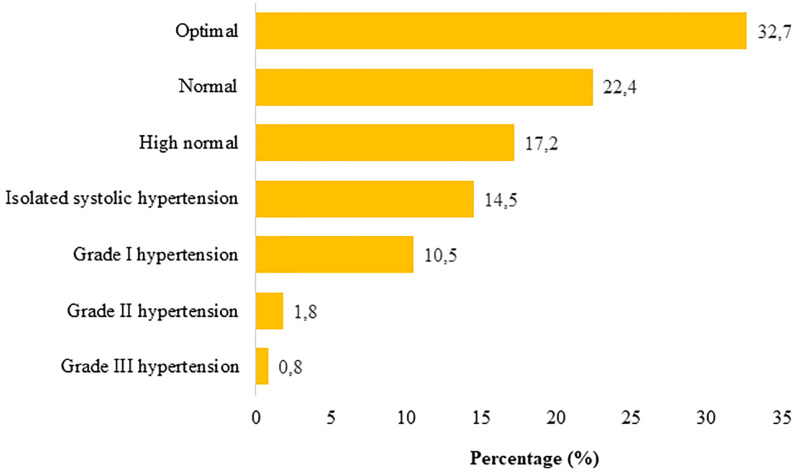
Blood pressure classification among migrants, Oujda, Morocco, 2021.

The prevalence of hypertension was 27.7% among the 495 participants recruited. The male/female ratio was 2.3, and the hypertensive migrants’ average age was 33.7±13.9 years. Among the hypertensive migrants, 91 (66.4%) were from Sub-Saharan Africa and 83 (60.6%) were in irregular legal status “Table 1”. According to clinical data, six (4.4%) participants had co-morbidity, and 69 (50.4%) were overweight or obese “Table 2”. Isolated arterial hypertension was present in 72 (52.5%) of the cases; 52 (37.9%) had hypertension stage I, nine (6.6%) had hypertension stage II, and four (2.9%) had hypertension stage III “Fig 1”.

### Bivariate analysis

Diabetes affected 19 (03.8%) of the participants. Following the bivariate analysis, the cut-off p-value was set at p≤0.05. According to the bivariate analysis, we identified eight risk factors for diabetes: 1. age (p = 0.003); 2. native Sub-Saharan African countries (p = 0.001); 3. legal status (p = 0.0005); 4. number of countries crossed greater than or equal to three (p = 0.002); 5. family history of diabetes (p = 0.001); 6. family history of hypertension (p = 0.002); 7. co-morbidities (p = 0.001); and 8. overweight/obesity (p = 0.01).

Hypertension affected 137 (27.7%) of the participants. Following the bivariate analysis, the cut-off p-value was set at p≤0.05. We identified nine factors associated with hypertension using bivariate analysis: 1. age (p<0.001); 2. marital status (p<0.001); 3. native of Sub-Saharan Africa (p<0.001); 4. living in Morocco for at least five years (p = 0.001); 5. legal status (p = 0.0006); 6. number of countries crossed (p<0.001); 7. alcohol consumption (p = 0.04); 8. family history of hypertension (p = 0.004); and 9. overweigh/obesity (p = 0.004) “Tables 1 and 2”.

### Multivariate analysis

After adjusting for the other variables, we identified family diabetes history (AOR of 5.36; CI% [1.23–23.28]) as a risk factor for diabetes among the migrant population in Oujda.

The age (AOR of 1.1; CI% [1.06–1.13]) and native Sub-Saharan African origin (AOR of 3.07; CI% [1.06–8.92]) as risk factors for hypertension after adjusting for the variables “Table 3”.

**Table 3:** Multivariate analysis (Odds ratio, *P-value*) of diabetes and hypertension risk factors among migrants in Oujda, Morocco, 2021.

	Multivariate analysis complete model for diabetes		Multivariate analysis complete model for hypertension	
	AOR [95%CI]	*p-value*	AOR [95%CI]	*p-value*
Age in years	1.05 [1.00–1.10]	0.03	1.10 [1.06–1.13]	<0.001
Partnered/Single	****	****	1.14 [0.59–2.20]	0.68
Native country Sub-Saharan Africa	0.95 [0.09–09.32]	0.96	3.07 [1.06–8.92]	0.03
Duration of stay in Morocco ≥5years	****	****	1.40 [0.75–2.60]	0.28
Legal status				
Irregular/regular	2.38 [0.20–27.30]	0.48	1.72 [0.61–4.78]	0.29
Asylum seeker/regular	0.61 [0.04–09.29]	0.72	0.64 [0.22–1.86]	0.42
Refugee/regular	1.58 [0.12–19.96]	0.72	0.26 [0.06–1.08]	0.06
Number of countries crossed ≥3	1.07 [0.14–07.98]	0.94	1.70 [0.82–3.52]	0.14
Alcohol consumption	****	****	0.55 [0.26–1.14]	0.11
Family history of diabetes	5.36 [1.23–23.28]	0.02	****	****
Family history of hypertension	1.20 [0.28–5.12]	0.79	0.39 [0.19–0.79]	0.008
Comorbidity	0.75 [0.11–4.81]	0.76	****	****
Overweigh/Obesity	1.61 [0.49–5.25]	0.42	1.42 [0.86–2.34]	0.16

AOR: Adjusted Odds Ratio, CI: Confidence Interval

Comorbidity stands for heart disease, dyslipidemia or chronic renal failure

## Discussion

Human migration has a significant impact on global population dynamics; it provides significant socioeconomic benefits, such as increased income and security, to those fleeing violence and persecution [13]. The current emergence of South-South migration, *i*.*e*. people moving between developing countries, contributes to inequalities and xenophobia, which are primarily the result of inadequate social and health systems. It is the source of negative health consequences for migrants, whether linked to communicable or noncommunicable diseases.

In our study, the prevalence of the Hepatitis C virus was 1% contrasting with the one obtained in Libya between 2013 and 2015, when the prevalence of HCV was 5.7% among 2557 Central African migrants and 8.1% among 4993 West African migrants [14]. However, it is consistent with the findings of a 2018 Italian study, which discovered that the prevalence of HCV was 0.9% among 11,084 Sub-Saharan African migrants [15, 16].

In our survey, the prevalence of HIV was 0.2% whereas the literature reports a higher prevalence of HIV among Sub-Saharan African migrants in Europe and the United States [17]. Indeed, Zencovich et al. found a 3% HIV prevalence among 634,958 people aged 15 and over from Zimbabwe, Burundi, Rwanda, Uganda, Zambia, and Chad who applied for permanent residence in Canada [18]. Migrants can contract HCV and HIV while traveling or in host countries as a result of rape, precarious living conditions, marginalization, and an unfavorable environment for adopting preventive behaviors [15, 19]. Some migrant populations engage in high-risk behaviors, such as having multiple sex partners, relying on sex workers, infrequently using condoms, and abusing alcohol and drugs.

According to previous epidemiological studies, migrants are more likely to develop diabetes complications, morbidities, and death [20]. Diabetes was diagnosed in 15% of 2,724 migrants aged 18 and up in Saudi Arabia in 2018 [21], and in 3.7% of 2058 migrants in Canada in 2003 [22]. In our survey, the prevalence of diabetes was 3.8%. In our study, diabetes in the migrant population was associated with a family history of the disease. This is consistent with previous research findings [21, 23]. Indeed, since 2007, pan-genomic studies have identified approximately fifty genetic markers linked to diabetes, particularly type 2 diabetes. These proteins have been linked to pancreatic beta cell dysfunction.

However, these genetic markers account for only 10% of type 2 diabetes heritability [24]. In our study, 27.7% of the migrants had hypertension, which is a major risk factor for cardiovascular disease.

This finding is consistent with a 2009 study of 226 migrants in Delhi, India, which found a hypertension prevalence of 20.3% [25]. The difficult living conditions during and after migration could explain the high prevalence of hypertension in our study.

An epidemiological study found a link between marital status and hypertension, with marriage protecting male health [26, 27]. This association could be explained by married men experiencing lower stress and anxiety levels, as well as eating a healthier and more closely monitored diet than single men. However, marital status was not associated with hypertension in our study’s multivariate analysis.

According to the literature, every ten kilograms of excess weight over the ideal weight causes a 3-mmHg increase in systolic blood pressure and a 2-mmHg increase in diastolic blood pressure [25, 27].

This link is stronger in cases of visceral obesity than in gynoid obesity. Obesity and being overweight are thought to be risk factors for diabetes and hypertension, but there was no evidence of this in our study.

In a study involving 2,459 Korean migrants, Lee CH et al. identified length of stay as a risk factor for hypertension [28]. This could be explained by loneliness in the host country, the absence of family, and the embarrassment of social inequity. Furthermore, not speaking the local language, insecurity, daily stress, irregular legal status, fear of being deported back to their country of origin, and failure to achieve the migratory project’s objectives, particularly after risking their lives on the path of migration to flee violence or persecution in their country of origin. During the current survey, migrants from Sub-Saharan Africa were more likely to present with high blood pressure than Syrians.

Differences in hypertension prevalence between Sub-Saharan migrants and Syrians could be attributed to differences in acculturation, diet, and ethnicity. The current survey results show that the migrant population has optimal blood pressure. Hypertension and aging have been linked. This finding is consistent with the 1948 Framingham Heart Study, which enrolled 5,000 people and discovered that blood pressure increased with age [10, 29]. Blood pressure may rise as people get older due to artery elasticity loss, long-term plaque accumulation, and an increase in the prevalence of heart and vascular diseases.

There are some limitations to this survey. During data collection, we encountered a prevarication bias, particularly in relation to economic and behavioral characteristics. Given the limited budget of the study, we could not focus on all sexually transmitted diseases, such as viral hepatitis B and syphilis. especially since the study was not only about screening the cases but also about confirming the diagnosis and ensuring the management of positive cases. Although the sampling suggests the presence of a selection bias where only migrants attending associations are included in the study, that is not entirely correct. Indeed, the associations of Oujda are very well known by migrants for the help and support they provide in terms of access to food, health care, and other basic life needs. Therefore, refugees, asylum seekers, undocumented migrants, and even newly arrived migrants frequent them.

## Conclusion

The prevalence of HCV, HIV, diabetes, and hypertension in our study was 1.0%, 0.2%, 3.8%, and 27.7%, respectively. Migrants seek better living conditions. They may become ill during the migratory journey or during their stay in the host country as a result of precarious living conditions and changes in lifestyle. Migration is a phenomenon that has the potential to exacerbate the growing and high burden of noncommunicable diseases, such as hypertension, particularly among lower socioeconomic classes, who may be more vulnerable to stress and unhealthy behaviors due to economic difficulties. A multifaceted approach is required, particularly for high-risk populations with multiple care needs. These findings also highlight the importance of implementing community awareness and mobilization activities for disease prevention and control among migrants.
